# The Effect of Gender, Age, and Nationality on the Personal Space Preferences in Children’s Hospitals among Iranian and German Children and Adolescents

**Published:** 2012-08-30

**Authors:** Sanaz Litkouhi, Masoud Geramipour, Sachli Litkouhi

**Affiliations:** 1 Department of Architecture, Payam Noor University, Tehran, IR Iran; 2 Allameh Tabatabaii University of Tehran, Tehran, IR Iran

**Keywords:** Demographic Statistics, Hospitals, Pediatric, Personal and Inter-personal Spaces

## Abstract

**Background:**

Patients in the therapeutic environment worry about their health and therapy; they are away from their usual social activities, and these entire put a great deal of stress on them, and delay their therapy by affecting their immune system and reducing their spiritual power.

**Objectives:**

The present study seeks to investigate the effect of gender, age, and nationality of children on their personal space preferences during hospital stay in children’s hospitals the dependent variable, i.e., personal space preference, was measured in hospital rooms with or without a barrier to separate children’s beds. (Barriers could be curtains or walls).

**Patients and Methods:**

The research method was descriptive survey research, and the participants included 120 Iranian children staying at Tehran children’s hospitals and 104 German children staying at Stuttgart’s children hospitals, who were chosen through stratified random sampling.(in all wards except infectious wards) The data was gathered through self report questionnaires and analyzed conducting categorical methods of Logic Log Linear Analysis and Logistic Regression methods using the SPSS software.

**Results:**

The results show that Iranian children, contrary to German children, prefer a space without any barrier, and that girls prefer closed spaces more than boys. Regarding the age, it was revealed that children and adolescents have nearly similar preferences. Finally, remarkable differences were found among groups with regard to their desired kind of barrier.

**Conclusions:**

Preferring a space without a barrier, which provides more chances for social interaction among individuals, shows that the majority of children prefer to have social interactions and contacts in the hospital that is in line with the results of similar studies. Last preference (separating the beds with curtains) was not generally favored by the respondents, which is in line with the results of similar studies in 2006.

## 1. Background

Patients in the therapeutic environment worry about their health and therapy; they are away from their usual social activities, and all of these put a great deal of stress on them, and delay their therapy by affecting their immune system and reducing their spiritual power [1]. Regarding children, this causes a much bigger problem as they have less compatibility with the environment, and less ability to analyze the problems. So, to reduce these problems, we can facilitate the child’s compatibility process with the therapeutic environment , and reduce the negative effects of the environment on their therapy process by designing the environment according to his/her desire, This is economically beneficial for the hospital as it reduces the period of the child’s hospital stay [2]. With regard to the population pyramid of Iran and the fact that a great number of children and adolescents are hospitalized before beings adults, and that the existing strategies in other countries, due to cultural differences, cannot be adopted exactly to be used in our country, it seems that examining the effects of hospital stay on children and adolescents, and also optimizing the present strategies is absolutely necessary.

## 2. Objectives

The current study is an attempt to examine the relationship between children’s demographic characteristics, including gender and age, as main criteria in spatial planning of therapeutic environment of hospitals, and also Iranian and German nationalities to find cultural differences regarding children’s spatial preferences.

## 3. Patients and Methods

### 3.1. Review of the Literature

Personal space means the limits within an individual’s surrounding environment which is determined by unseen boarders. One is irritated by the presence of anybody in this particular space. This space is not necessarily round in shape, and does not expand in the same extend from all its dimensions [3]. Gafman proposed another definition for personal space, i.e., “the space around a person to which the entrance of another person is considered as violating her/his limits, which causes irritation, and even abandonment”. One of the most remarkable views regarding personal space belongs to Hall, who describes it as proxemics when examining the relationship between humans and space in his book, called “The Hidden Dimension”. In his classification of spaces, personal space distance occurs in the second part, after the intimacy distance. In one of the chapters, entitled “Distance in Men”, regarding the formless space, Hall notes that, “Not only do birds and mammals have a land that they occupy and protect it against their own kind, but they also keep some fixed distances among themselves. Hydger has classified these distances as runaway distance, critical distance, personal distance, and social distance. Humans also have similar ways to keep distance from others. The runaway distance and the critical distance, except for a few cases, have been deleted from human reactions. Yet, the personal and social distance clearly do exist. Now the questions are: How many different distances between humans exist? And how do they distinguish one from the other?” According to Hall, humans consider space not as something static based on the linear perspective of Renaissance, but something dynamic. He maintains that an incorrect understanding of space can be due to two main factors: One is that we always try to find one specific cause for any effect; the other factor is that we consider the beginning and end of human limitations the same as his/her physical body [4]. One of the most important indices of determining the distance between individuals is their cultural characteristic [5]. In this regard, a number of inter-cultural studies have been carried out on the psychological and social characteristics of people in different cultures and countries, some of which have specifically concentrated on the factors affecting personal distance. The results indicate that there are fundamental differences among cultures regarding the application of personal distance, the distances they keep, the form of the territory, and their interpretations of intimate behavior. Among other effective indices we can refer to the perception of space, interpersonal behavior, and the extend of individualism: *Space:* Dolphin has studied the use of space, as another aspect of human interaction, in different cultures. The resultant hypothesis is that the use of space by an individual is a message, transferring a meaning to others around him/her. One’s perception of space and its use is directly related to his/her culture, which is discussed in Dolphin’s article entitled “Personal space Variables in Cultural Interactions”. According to Dolphin, culture is more than an individual’s hometown, and the age, gender, relationship, environment, and ethnic group are all important in one’s communicative interaction [6].

### 3.2. Interpersonal Behavior: Immediacy and Expressiveness

In discussing cultural influences on subjective wellbeing, Triandis has proposed the concept of “Person-Environment Fit “Having a personality which matches the values of the overarching culture should increase subjective wellbeing, while a mismatch will decrease it [7]. Another factor in determining the intimacy and distance between individuals is interpersonal behavior. Intimate behaviors transfer sociability, closeness, and readiness for communication. They show willingness against avoidance, and intimacy against abandonment. Some examples of intimate behaviors are smiling, touching, eye contact, close distance, and voice changes. Some researchers consider these behaviors as “meaningful”. The cultures in which there is a lot of interpersonal intimacy and closeness are called” Contact Cultures” because people in these countries stand close to each other, and usually touch each other. People in Less Contact Cultures stand within a distance from each other and occasionally touch each other. It is interesting that Contact Cultures are usually in countries with warm weather (e.g., Arabian countries, countries in Mediterranean zone such as France, Greece, Italy, Spain, East Europe) , and Less Contact Cultures in countries with cold weather ( e.g., parts of the north of Europe such as Scandinavian countries, Germany, England, Russia, and also Japan). The geographical differences include energy level, climate, and body metabolism. The culture of cold areas is clearly work-centered and “cold” regarding interpersonal relations, whereas the culture in warm areas is relation-centered and “warm” [8].

### 3.3. Individualism

One of the fundamental cultural differences is the degree of individualism as opposed to collectivism. This cultural dimension determines the way people live with each other (alone, in a family, or in a tribe), their values, and their relations. Western culture is an individualistic culture, whereas eastern culture emphasizes homogeneity and relationship among people and between people and nature. Those in favor of individualism consider it as the basis of liberalism, democracy, free will, and economical motivation, and a protective factor against cruelty. On the other hand, individualism has been criticized for alienating people from each other, causing loneliness, egoism, and proud. People in individualistic cultures isolate from each other whereas in collectivist cultures, they have relations with each other, and as a result their work, entertainment, life, and rest all happen at a closer distance from each other. [9]. People in individualistic cultures smile more than people in collectivist cultures. This can be due to the fact that individualistic people consider themselves responsible for their own relations and happiness, whereas in collectivist cultures people consider accepting norms as the most important value, and the individual or interpersonal happiness as the secondary value [10]. In the other hand individualists frequently think of self-reliance as being able to pursue their own goals while for collectivitists self-reliance means not being a burden on one’s in-group [11]. Industrialized nations such as United States, England,… are regarded as individualistic while developing regions such as Africa, Chine, typically have traditional values and are collectivistic [12]. Another part of studies regarding personal space include those concentrating on specific environments. For example, a study on the university and high school environments reveals the effect of gender and age on personal space in these environments. Tennis and Dabbs indicated that university students as compared to high school students tend to have more personal space [13]. Other studies show that males prefer larger personal space than females. Lomranz and his team studied the effect of gender and age on children’s personal space. In this study, some differences were found between the preferences of 3 and 5-7 years old children with regard to personal space. It was also revealed that girls, in comparison with boys, stand closer to each other [14]. In a study, regarding the effect of class organization on personal space preferences of students, Brody and Zimmerman concluded that the class organization has an effect on personal space preferences of students, and that children in open- area classes prefer closer personal space than traditional classes [15].

Another part of the studies has concentrated on the privacy in therapeutic environment, the debate over the role of privacy in patient well-being and particularly in the context of the architecture of the patient room, has a long history within the history of the hospital [16]. As privacy and personal control lighting levels, spatial props, view, and so on believed to influence patient outcomes [16]. Privacy (in hospital) is an umbrella condition or state consisting of the sum total of five interrelated dimensions: auditory privacy (sound), visual privacy (sight), tactile privacy (touch),spatial privacy (personal space) and olfactory privacy (smell) [17]. In the other hand cognitive and spatial control is highest when the patient and family are in space of their own, undisturbed by outsiders, and where persons unfamiliar to the patient and family do not share the same zone of personal space [18].

Geden and Begeman who studied the personal space preferences during hospital stay. This study reveals some differences personal space preferences of hospitalized adults. Here, the preferred personal space in interacting with nurses, doctors, and family members and the effect of gender and age on personal space preferences has been studied. The results show that the personal distance of patients from their family members is smaller than the distance from doctors and nurses, and that the age and gender variables normalize this relationship [19].

Another research indicates that children naturally tend to establish social relations, and that forming peer groups, especially for adolescents in the hospital, is of utmost importance [20]. The NAWCH study, done in 2001, concludes that it is absolutely crucial to pay attention to the social needs of adolescent patients, emphasizing the appropriate design of the hospital to promote social interactions of children and adolescents.

Another remarkable research in this case has been done by Devlin and Blumberg, studying the views of 54 boys and 46 girls, aged between 12 to 14, about physical designing and visual characteristics of hospitals. They also studied the adolescents’ preferences regarding the existence of solitary, hospital space, entertainments and leisure activities, public spaces, and visiting hours. The study indicated that in hospitals, the sense of personal control is in danger, but through designing we can help patients to specify their personal boarders, and feel that they a private space. This is specifically important for adolescents considering their specific characteristics regarding their age. In this study, the adolescents were required to indicate the items which they considered important in having privacy in hospital; 96% of the adolescents mentioned having private bath, 89% having single room, and the possibility of closing the door of the room, which indicate their need for privacy and solitude. Studies show that this sense extremely changes in adolescents from the age of 11 to 13. 82% of adolescents preferred to wear comfortable clothes which covered all their body. Even some of them liked to bring their own pajamas to the hospital, which all indicate that adolescents tend to have privacy and control, and a sense of individuality in their peer group. Regarding their sexual characteristics, adolescents are shy, and do not like the short clothes of the hospital which are open in the back, and aggravate these negative feelings. Strategies, such as pulling curtains around the beds to provide privacy, were only accepted by 28% of adolescents [1].

### 3.4. Methodology

The present study is a descriptive- analytic (non- experimental) research regarding the method of data gathering. It is also a demographic research. Moreover, considering the purpose of the research, this study is an applied research. Measurement Instrument: To obtain children’s viewpoints and preferences based on research objectives, a questionnaire for children was constructed. The questionnaire was easy enough to understand and to answer by children especially for sick children because of their special conditions. This questionnaire were developed in two Iranian and Deutsch language formats for sick children with 2 major questions about barriers and type of them(curtain, wall or nothing) and demographic characteristics of the children(gender, age group, nationality). There were some considerations in constructing questionnaire due to children’s characteristics: The first was limiting the number of questions and the second one was designing questionnaire graphically. Due to children’s problems in imagining space and also their lack of ability in responding to questions orally, the answers of graphic-based questionnaires have more reliability than other kind of questionnaires.

The face validity and content validity of the questionnaire used in the interview were confirmed by child psychology experts before its implementation. Sample: Considering the limitation of doing the research in all cities of Iran and Germany, the research population is delimited to the children hospitals in Tehran and Stuttgart. The sample was selected from different wards of children hospitals (except infectious wards). The sample under study included 106 girls (47.3%) and 118 boys (52.7%). the sample included 120 (53.6%) Iranian children, hospitalized in the children’s hospitals of Tehran, and 104(46.4%) German children hospitalized in the hospitals of Stuttgart. The sample size estimated based on Cochran formulae with accepting error magnitude of 0.1. Stratified random sampling was applied to select the children in which at first the city partitioned into four geographical direction (North, South, West and East) and then about %70 of the hospitals randomly selected from each direction and at last in each hospital children were randomly and proportionally selected based on sample size Also, the sample included 135 (60.3%) children aged 6 to 11, and 89 (39.7) adolescents aged 12 to 16.The data was gathered in 2006-7.

## 4. Results

In order to investigate the effect of gender, nationality, and age group, as independent variables, on the space preferences during hospital stay, the dependent variable was studied from two approaches. First, to examine the exact kind of the barrier (wall or curtain) or without a barrier, Logit Log Linear Analysis was carried out. Then, the dependent variable at two levels, with barrier and without barrier, collapsed, and the effect of independent variables on it were examined using Logistic Regression. The results of the Logit Log Linear Analysis, to investigate the corresponding relationship between the independent variables of gender, nationality, and age group, and space preferences (wall, curtain, or without a barrier) as indicated in Table 1 a model with an appropriate Goodness of fit.

**Table 1:** Parameter estimates of Logit Log Linear

	**Estimate**	**Standard Error**	**Z**	**Significance level**
Curtain Choosing	-1.054	.359	-2.933	.003
Wall Choosing	-.611	.429	-1.425	.154
No barrier Choosing	0(A)			
Curtain, 6-11	.564	.341	1.653	.098
Curtain, 12-16	0(A)			
Wall, 6-11	-.949	.449	-2.113	.035
Wall, 12-16	0(A)			
No barrier, 6-11	0(A)			
No barrier, 12-16	0(A)			
Curtain, Iranian	-.684	.318	-2.151	.031
Curtain, German	0(A)			
Wall, Iranian	-.980	.452	-2.166	.030
Wall, German	0(A)			
No barrier, Iranian	0(A)			
No barrier, German	0(A)			
Curtain, Girls	1.037	.316	3.280	.001
Curtain, Boys	0(A)			
Wall, Girls	.226	.448	.503	.615
Wall, Boys	0(A)			
No barrier, Girls	0(A)			
No barrier, Boys	0(A)			

Thus, other competent models could not overshadow the present model considering the ease of interpretation and goodness of fit of the data (χ2 (8) = 9/23, P = 0/321).

The results of Table 1 indicate that, in general, the probability of choosing a curtain or a space without a barrier is significantly less than what is expected (B = -1/05, P < 0/01), whereas the probability of choosing a wall or a space without a barrier does not have significant difference (B = -0/61, P > 0/05). Therefore, on the whole, without considering the mentioned demographic characteristics, it is revealed that curtain is less favored by children. The probability of choosing curtain by children aged 6 to 11, does not have any significant difference with the probability of the same choice with children aged 12 to 16 (B = 0/61, P > 0/05). This means that age groups cannot have any effect on choosing curtains. The probability of choosing a wall by children aged 6 to 11, compared with the probability of the same choice by children aged 12 to 16, is significantly less than what is expected (B = -0/95, P < 0/05). Also, the probability of such a choice, regarding a space without a barrier, by the two age groups is less than what is expected. We can conclude that using a wall, compared with other choices, is not desirable for separating children aged 6 to 11. The probability of choosing a curtain by Iranian children, compared to the same choice by German children, is significantly less than expected (B = -0/98, P < 0/05). Moreover, the probability of such a choice, compared with choosing a wall, by Iranian and German children is less. Thus, Iranian children prefer curtains less than German children. The probability of choosing a curtain by girls is significantly more than boys (B = 1/04, P < 0/01), but there is no difference between boys and girls in choosing wall (B = 0/448, P > 0/05). Neither is there any difference between boys and girls in choosing wall or an open space. Thus, if we have to use curtains, it is better to use it for separating girls.

Also as showed in Table 2 , the Backward Ward Logistic Regression analysis, used for studying the relationships of the independent variables of nationality, age group, and gender, with spatial preferences (both with or without a barrier), at two stages, provided a fit model.

**Table 2:** Parameter estimates of Logistic Regression analysis

**Model parameters**	**Estimates**	**Standard Error**	**Wald statistics**	**DF**	**Significant Level**	**EXP (B)**
First Stage						
Nationality (Iranian)	-.733	.285	6.599	1	.010	.480
Age Group (6-11)	.101	.294	.118	1	.731	1.106
Gender (Girl)	.820	.283	8.414	1	.004	2.270
Constant	-.269	.303	.786	1	.375	.764
Second Stage						
Nationality(Iranian)	-.716	.281	6.503	1	.011	.489
Gender (Girl)	.809	.281	8.307	1	.004	2.245
Constant	-.211	.252	.702	1	.402	.810

Also the results of Hosmer and Lemeshow Test indicate the appropriate fitness of the observed data to the expected data (χ^2^ (2) = 0/790, P = 0/674).

As indicated in Table 2 the results of the parameter estimates for the final stage of the Logistic Regression model reveal that there is a statistically significant relationship between the two variables of nationality (B = -0/72, P < 0/05) and gender (B = 0/81, P < 0/01) , and two dimensional space preferences (i.e., with barrier or without barrier). The age group variable did not show any significant relationship at the first stage of Logistic Regression analysis, and exited from the model (B = 0/10, P > 0/05). These relations mean that Iranian children prefer open spaces, without a barrier, more than German children. This preference is vice versa for German children, that is, being an Iranian reduces the chance of choosing a space with a barrier by 49%.Moreover, girls prefer spaces with barrier more than boys, that is, and being a girl increases the chance of choosing a space with a barrier by 2/24%. Finally, lack of any relationship between age groups and their preferences show that is no difference between the preferences of children and adolescents.

## 5. Discussion

Reviewing the views of the children and adolescents indicate that, regarding their private space in hospitals, they prefer a space without a barrier, a wall, and a curtain respectively. Totally wall was not preferred by children and in most situations they were indifferent in choosing wall as barrier. Preferring a space without a barrier, which provides more chances for social interaction among individuals, shows that the majority of children prefer to have social interactions and contacts in the hospital, which is in line with the results of the similar research. For example, Platt based on a similar study, notes that children naturally tend to establish social communication, and the research done in 1993 indicates that it is very important for adolescents to form peer groups in the hospital. Also, the research done by NAWCH in 2001 emphasizes the importance of social needs for adolescent patients. This has been taken into consideration in designing children’s hospitals.

Regarding the effects of nationality on the children’s preferences, it can be concluded that Iranian respondents preferred spaces without a barrier more that German respondents and that boys preferred this more than girls. This is also in line with the results of similar studies. Yet, there was no difference between children and adolescents in this regard. The second preference in the responses was to have a wall between the patients, which was more favored by adolescents. This shows that adolescents tend to have privacy more than children. Other similar researches done in 1980 and 2003 conclude that adolescents face a lot of problems regarding privacy in hospitals, and that ideal therapeutic environments are those which have designed single-bed rooms as well as spaces for establishing social interactions. In another research done in 2006, regarding hospital wards, two systems, i.e., open and separated rooms, were studied. The advantages of separated room were quietness, more comfortable sleep, more comfortable stay of parents, having private bath and TV, and the disadvantage was the lack of social interactions. The last preference of the respondents was having a curtain around the patients’ bed. Girls more than boys preferred to have curtains around their beds, which seems to be logical with regard to cultural issues. However, this last preference (separating the beds with curtains) was not generally favored by the respondents, which is in line with the results of similar studies of Delvin and Blumberg in 2006.
